# Diversity of *Anopheles* mosquitoes in Binh Phuoc and Dak Nong Provinces of Vietnam and their relation to disease

**DOI:** 10.1186/1756-3305-7-316

**Published:** 2014-07-09

**Authors:** Chung Thuy Ngo, Gregor Dubois, Véronique Sinou, Daniel Parzy, Hong Quang Le, Ralph E Harbach, Sylvie Manguin

**Affiliations:** 1 Institut de Recherche pour le Développement (IRD), LIPMC, UMR-MD3, Faculté de Pharmacie, F-34093 Montpellier, France; 2 National Institute of Veterinary Research, Hanoi, Vietnam; 3 UMR-MD3/IRBA, Faculté de Pharmacie, Aix-Marseille Université, F-13385 Marseille, France; 4 Military Preventive Medicine Centre, Ho Chi Minh City, Vietnam; 5 Department of Life Sciences, Natural History Museum, London SW7 5bD, UK

**Keywords:** *Plasmodium*, *Wuchereria bancrofti*, *Anopheles*, Vietnam, Real-time PCR

## Abstract

**Background:**

Human malaria is still a burden in Dak Nong and Binh Phuoc Provinces in south-central Vietnam that border Cambodia. Several *Anopheles* species that transmit human malarial *Plasmodium* may also transmit *Wuchereria bancrofti*, the nematode that causes Bancroftian lymphatic filariasis. The objective of this study was to investigate the role of *Anopheles* species in the transmission of these two pathogens in the two highly malaria endemic provinces of Vietnam.

**Methods:**

*Anopheles* mosquitoes were collected in Dak Nong and Binh Phuoc Provinces in November and December of 2010 and 2011. Human landing catches, paired collections on human and buffalo, and resting captures were made with mouth aspirators. Collections were also made with light traps. Morphological and PCR-based methods were used to identify the species. Real-time PCR was used to detect *Plasmodium* species and *W. bancrofti* in individual mosquitoes.

**Results:**

Twenty-four *Anopheles* species were identified among 797 captured mosquitoes. *Anopheles dirus* was found in both provinces and was the predominant species in Binh Phuoc Province; *An. maculatus* was the most prevalent species in Dak Nong Province. *Anopheles minimus* was collected only in Binh Phuoc Province. Some specimens of *An. minimus* and *An. pampanai* were misidentified based on morphology. Four specimens of *An. scanloni* were identified, and this is the first report of this species of the Dirus Complex in Vietnam. Two females, one *An. dirus* and one *An. pampanai*, collected in Binh Phuoc Province were infected with *P. vivax*, for an overall infection rate of 0.41% (2/486): 0.28% for *An. dirus* (1/361) and 20% for *An. pampanai* (1/5). No mosquitoes were found to be infected with *P. falciparum*, *P. knowlesi* or *W. bancrofti* in either province.

**Conclusion:**

A diversity of *Anopheles* species occurs in Dak Nong and Binh Phuoc Provinces of Vietnam, several of which are considered to be actual and potential vectors of malarial protozoa and microfilariae. It is highly likely that two of the species, *An. dirus* and *An. pampanai*, are active in malaria transmission based on the detection of *P. vivax* in females of these species. This is the first report of *An. scanloni* in Vietnam.

## Background

Despite a decline in human malaria cases in Vietnam, malaria transmission continues in the forested areas of the southern and central provinces [1-5] where it is a significant public health and economic problem [6]. Several factors contribute to the maintenance of malaria transmission in this region of Vietnam, including a high density of the main vector *Anopheles dirus*[3,7,8], migration of people from malaria endemic areas [9] and failure to use preventive measures while working in the forest [10]. Moreover, use of insecticides and prophylactic drugs has resulted in resistance in some *Anopheles* species [11-13] and malarial parasites [14,15]. Furthermore, the monkey malarial protozoan *Plasmodium knowlesi*[16-21] has been found in both humans [7,22] and *Anopheles* mosquitoes in Vietnam [7,23]. Marchand *et al.*[7] reported co-infection of *P. falciparum*, *P. vivax* and *P. knowlesi* in an *An. dirus* female, indicating that *P. knowlesi* is most likely transmitted by the same primary vectors of *P. falciparum* and *P. vivax*. Hence, malaria caused by *P. knowlesi* may represent an additional challenge for malaria control in forested areas where *An. dirus* and the natural hosts of *P. knowlesi* (macaque monkeys) co-occur, and zoonotic transmission of *P. knowlesi* could continue in the absence (or elimination) of *P. falciparum* and *P. vivax*.

*Anopheles* vectors involved in the transmission of *Plasmodium* parasites may also transmit *Wuchereria bancrofti*, the nematode that causes 90% of the lymphatic filariasis cases worldwide [24]. The co-occurrence of malarial and filarial parasites has been reported in endemic areas [25-28]. Unlike malaria, filariasis, a neglected disease, is rarely fatal but severe morbidity, including disabilities and adverse economic consequences due to disfigurement of limbs and male genitals (elephantiasis and hydrocele, respectively), occurs in 40% of infected individuals [29,30]. Bancroftian lymphatic filariasis occurs in the southern and central provinces of Vietnam [31,32], but little information is available about the co-infection of the microfilariae and malarial parasites in *Anopheles* mosquitoes. Even though Vietnam is now in the pre-elimination phase for the control of lymphatic filariasis [33], it is important to obtain additional information on the prevalence and transmission of this disease to better define its distribution in the country.

Binh Phuoc and Dak Nong Provinces are located in forested areas of south-central Vietnam. These two neighboring provinces share the border with Cambodia and are recorded as having the highest burden of malaria nationwide [14,34,35]. Although resistance of *P. falciparum* to the anti-malarial drugs chloroquine, sulfadoxine-pyrimethamine and mefloquine has been reported in these provinces [14,15], no information concerning the transmission of malarial and filarial parasites is available.

To understand the role of *Anopheles* species in the transmission of *Plasmodium* and *W. bancrofti*, and document the diversity of the anopheline mosquitoes in Binh Phuoc and Dak Nong Provinces, we conducted medical entomological surveys in these provinces during 2010 and 2011.

## Methods

### Study area

*Anopheles* females were collected in two communes, Bu Gia Map in Binh Phuoc Province (11° 45′ N, 106° 43′ E) and Dak Ngo in Dak Nong Province (11° 59′ N, 107° 42′ E). Temperature in these provinces is around 25°C during the day and falls to 7–9°C at night; the rainy season extends from May to October and the dry season from November to April. The climate is favorable for agriculture, especially coffee, pepper and rubber. Crops of coffee, pepper or cashew nuts are usually grown around houses. The villages are surrounded by cassava, corn and rice fields, and are fringed by forest. The majority of income is from agricultural production. Every year, during harvest time, workers from neighboring regions come to work in the fields; hence, high population movement occurs in these areas. The wood houses are mostly built directly on the ground, but houses near forest or forest fringe are built on stilts. During harvest periods, people live in huts that provide little protection from mosquitoes.

Dak Ngo commune covers an area of 16,624 ha and has a population of 8,478 (in 2012), with ethnic minorities accounting for 65% of the residents. Annual incomes are low, 58% of households are poor. Transportation is difficult, especially during the wet season, because most roads are unpaved. Plantations have replaced most of the forest. In 2011, 24 malaria cases were reported in Dak Ngo, including 14 caused by *P. falciparum* and 10 due to *P. vivax* (data provided by the Dak Ngo community health station).

Unlike Dak Ngo, the roads in the Bu Gia Map commune are mostly paved and the area is mostly surrounded by primary forest, which includes the Bu Gia Map National Park. This commune covers an area of 2,330 ha, has 3,704 inhabitants (in 2009) and is a tourist destination. In 2011, 266 malaria cases were treated, 124 caused by *P. falciparum*, 138 due to *P. vivax* and four were mixed infections of *P. falciparum* and *P. vivax* (data provided by the Bu Gia Map community health station).

### Field collections and morphological identification of mosquitoes

Four methods were used to capture mosquitoes: outdoor human landing catches, paired collections on human and buffalo and resting captures using mouth aspirators, and light trap collections. All collections were conducted from 17:00 until 07:00. The period of collections was the same during 2010 and 2011, during the change from the rainy to the dry season (November and December) when mosquito populations had peaked and a greater number of pathogen-infected females was anticipated. [It should be noted that limited and difficult access to the study sites prevented collections from being made during most of the rainy season.] Mosquitoes captured each hour by each method were retained in separate cups and labelled accordingly. Specimens were identified the following morning using the keys published by the National Institute of Malariology, Parasitology and Entomology [36,37], killed, divided into two parts, head-thorax (with legs and wings) and abdomen, and each part was stored separately at -20°C. The specimens were shipped on dry ice to the IRD laboratory in France and stored at -80°C until further use.

In the Dak Ngo commune, mosquitoes were collected at six sites during nine nights in November 2010. During November and December 2011, no *Anopheles* mosquitoes were collected at these six sites; hence, an additional seventh collecting site was added where a few *Anopheles* were collected. These collections began at 17:00 and ended at midnight. The paired collections on human and buffalo were made at two sites by two collectors per site, one person performed human landing catches and another person made either resting captures or paired collections.

In the Bu Gia Map commune, human landing catches and light trap collections were made at three sites during 11 nights. Collections were made from 17:00 to 20:00. Three or four collectors seated about 10 m apart worked at each site. Two light traps were placed near the collection site.

### Ethical statement

The Military Preventive Medicine Centre, Ho Chi Minh City (Vietnam) organized the field study and obtained all necessary permits. Vietnam People’s Army Department of Military Medicine approved the study. Mosquito collections were done with the approval of the head of each village and the owner and occupants of the houses where mosquitoes were collected. Mosquito collectors gave their consent and were treated free of charge for malaria presumed illness throughout the study in accordance with the national drug policy of Vietnam.

### DNA extraction

Genomic DNA was extracted from the head-thorax portions of mosquitoes using the Qiagen DNeasy Kit (Qiagen Ltd., Sussex, England). The manufacture’s protocol was followed to obtain a final volume of 100 μl of extracted DNA in TE buffer. Extracted DNA was divided and stored in separate tubes at -20°C for sequencing and parasite detection.

### Molecular identification of *Anopheles* species

Following morphological identification, DNA was extracted from *Anopheles* specimens belonging to groups and complexes of sibling species for which specific PCR identification methods are available, including the species of the Minimus Complex and closely related species such as *An. aconitus*, *An. pampanai*, *An. varuna*[38], the Dirus Complex [39], and the Maculatus Group [40]. Tfi DNA polymerase (Invitrogen, France) at 5 units/μl was used in all PCR assays. The PCR assay of Walton et al. [39] for the Dirus Complex was modified as follows: the final concentration of DMSO was reduced from 4% to 3.2%, one unit of Tfi DNA Polymerase was substituted for 0.25 unit DNA Polymerase and 2 μl of DNA template was used instead of 0.5 μl. The number of amplification cycles was also increased, from 32 to 35. Positive controls for *An. minimus, An. harrisoni, An. aconitus, An. pampanai, An. varuna, An. dirus, An. scanloni, An. baimaii, An. maculatus* and *An. sawadwongporni*, available at LIPMC (IRD, Montpellier, France), were used as references.

### Sequencing

Fragments of the mitochondrial genes (cytochrome oxidases 1 and 3, cox I and III) of *Plasmodium*, and fragments of the mitochondrial DNA (Cytochrome Oxidase subunits 1 and 2, COI and COII) and ribosomal DNA (Domain 3, D3, and Internal Transcribed Spacer 2, ITS2) of *Anopheles* mosquitoes were amplified and sent out for sequencing (Millegen, Labege Cedex, France). The fragments of cox III for *P. falciparum* and cox I for *P. vivax* were amplified using the PCR assay of Cunha et al. [41].

For *Anopheles* species, the D3 and ITS2 fragments of rDNA were amplified using the pair of primers of Sharpe et al. [42] and Beebe et al. [43], respectively. The COI and COII mitochondrial genes were amplified using the pair of primers described in Simon et al. [44] and Yang et al. [45], respectively. Standard protocols were used for all PCR amplifications. Each 50 μl PCR reaction contained 5 μl of 10X AccuPrime Pfx reaction mix, 1.5 μl of each primer (10 μM), 0.4 μl of AccuPrime Pfx DNA polymerase (2.5 units/μl) and 4 μl of template DNA. Thermal cycling conditions consisted of an initial denaturation step at 95°C for 2 min, then 40 cycles each consisting of denaturation at 95°C for 15 s, annealing for 30 s at 50°C for COI, COII and ITS2 and at 63°C for D3, and extension at 68°C for 45 s with final extension at 68°C for 10 min. The PCR products were kept at 4°C and checked by electrophoresis in 1.5% agarose gel, containing gel red 1X at final concentration.

Successfully amplified products were purified using the Nucleo Spin DNA purification kit (Macherey Nalgen, Germany). Direct sequencing of the products in both directions was done with an ABI 377 automated sequencer (PE Applied Bio Systems, Warrington, England).

### Detection of *Plasmodium* species and *Wuchereria bancrofti* using real-time PCR

LightCycler system 480 (Roche Applied Science, Penzberg, Germany) was used for the real-time PCR detection of sporozoites of three *Plasmodium* species (*P. falciparum, P. vivax* and *P. knowlesi*) and the presence of *Wuchereria bancrofti* in the head-thorax portions of the mosquitoes*.* The sequences of the primers and probes used to amplify the target sequences were described by Rao et al. [46,47] and Divis et al. [48], and are listed in Table 1. The Taqman probes were labelled with the reporter dyes FAM, HEX or Cy5 at the 5′ end, and the quencher dyes Black Hole Quencher 1 (BHQ1) and 2 (BHQ2) were added to the 3′ end (detailed in Table 1). Primers and probes were commercially synthesized by Eurogentec (Angers, France). Extracted DNA used as positive controls for *P. falciparum* and *P. vivax* was provided by DP, and that used for *Wuchereria bancrofti* was provided by Dr. J. Pothikasikorn (Mahidol University, Bangkok, Thailand).

**Table 1:** **Sequences of primers and probes used for the detection of *Plasmodium *** **species and *W. bancrofti *** **using multiplex real-time PCR**

**Name**	**Sequence (5′ - 3′)**	**Target**
Plasmo 1	GTTAAGGGAGTGAAGACGA TCAGA	*Plasmodium* sp. and *P. knowlesi*
Plasmo 2	AACCCAAAGACTTTGATTTC TCATAA	
Plasprobe	FAM-ACCGTCGTAATCTTAACCATAAACTATGCCGACTAG-BHQ1	
Pk probe	HEX-CTCTCCGGAGATTAGAACTCTTAGATTGCT-BHQ1	
FAL_F	CTT TTG AGA GGT TTT GTT ACT TTG AGT AA	*Plasmodium falciparum*
FAL_R	TAT TCC ATG CTG TAG TAT TCA AAC ACA A	
FAL_PB	FAM - TGT TCA TAA CAG ACG GGT AGT CAT GAT TGA GTT CA - BHQ1	
VIV_F	ACG CTT CTA GCT TAA TCC ACA TAA CT	*Plasmodium vivax*
VIV_R	ATT TAC TCA AAG TAA CAA GGA CTT CCA AGC	
VIV_PB	HEX - TTC GTA TCG ACT TTG TGC GCA TTT TGC - BHQ1	
LDR1_F	ATT TTG ATC ATC TGG GAA CGT TAA TA	*Wuchereria bancrofti*
LDR2_R	CGA CTG TCT AAT CCA TTC AGA GTG A	
WB_PB	Cy5 - ATC TGC CCA TAG AAA TAA CTA CGG TGG ATC TCT G - BHQ2	

The real-time PCR mix was prepared using the Platinum Taq polymerase Kit for each PCR run. Total volume (20 μl) of the mix containing (in final concentration) 1X PCR buffer, 4 mM MgCl_2_, 200 μM dNTP mixture, 0.225 μM of each primer, 0.1 μM of probe, 0.5 Unit Platinum Taq polymerase and 2 μl of DNA template was used to fill a 384-well microplate (Roche Applied Science, Penzberg, Germany). Singleplex real-time PCR was preceded by positive and negative samples to detect the separated target and check the repeatability of the method.

Multiplex assays were designed to simultaneously detect several parasite species, e.g. *Plasmodium* species, *P. vivax* + *W. bancrofti* and *P. falciparum* + *P. knowlesi*. Amplification consisted of one cycle of denaturation at 95°C for 15 min followed by 45 cycles at 95°C for 20 s, 60°C for 1 min and 40°C for 30 s. Analysis of the singleplex and multiplex data was performed with the LightCycler 480 software (Roche Applied Science, Penzberg, Germany). PCR grade water was used as no-template control. DNA from *W. bancrofti*, *P. falciparum* and *P. vivax* served as positive controls, and DNA extracted from human blood and *An. dirus* free of *Plasmodium* and *W. bancrofti* served as negative controls. All real-time PCR assays were carried out in duplicate, and samples that did not produce fluorescence before the threshold of 40 cycles (Ct) were considered negative. Questionable samples with a single positive well were retested. Specimens that gave a positive signal for the presence of parasites in the real-time PCR before 40 Ct were double checked using conventional PCR developed by Cunha et al. [41] for the detection of *P. falciparum* and *P. vivax*.

## Results

### Identification of *Anopheles* mosquitoes

A total of 797 *Anopheles* females were collected in Dak Nong and Binh Phuoc Provinces during November and December of 2010 and 2011. Morphological identifications showed that the specimens represented 20 species, including major malaria vectors of the Dirus Complex and the Funestus and Maculatus Groups (Table 2).

**Table 2:** ***Anopheles *** species collected in Dak Nong (*) and Binh Phuoc (**) Provinces

**Complex/group**	**Taxa**	**Morphological identification**	**Molecular identification**
Annularis Group	*An. philippinensis*	5	-
Asiaticus Group	An. asiaticus*	1	-
Barbirostris Group	An. barbirostris**	1	-
	*An. barbumbrosus*	23	-
	An. campestris*	5	-
**Dirus Complex**	*An. dirus*	390	384
	An. scanloni**	0	4
	Unidentified	0	2
**Funestus Group**	An. aconitus**	1	1
	An. minimus**	83	78
	An. pampanai**	0	5
	An. jeyporiensis**	5	-
Hyrcanus Group	An. crawfordi*	49	49
	*An. nitidus*	2	-
Kochi Group	*An. kochi*	18	-
Lindesayi Group	An. gigas*	24	-
**Maculatus Group**	*An. maculatus*	175	152
	*An. sawadwongporni*	0	16
	An. rampae**	0	6
	Unidentified	0	1
	An. karwari**	1	-
Subpictus Group	An. indefinitus**	1	-
	*An. subpictus*	4	-
	An. vagus*	2	-
Tessellatus Group	An. tessellatus*	4	-
Umbrosus Group	An. umbrosus*	3	-
**Total**		**797**	

*Species collected in Dak Nong Province only; **Species collected in Binh Phuoc Province only; No asterisk indicates species present in both provinces. Groups that include major malaria vectors are in boldface type. Molecular identification to species level is based on PCR assays available (only) for the Dirus Complex and the Funestus, Hyrcanus and Maculatus Groups.

**Figure 1:**
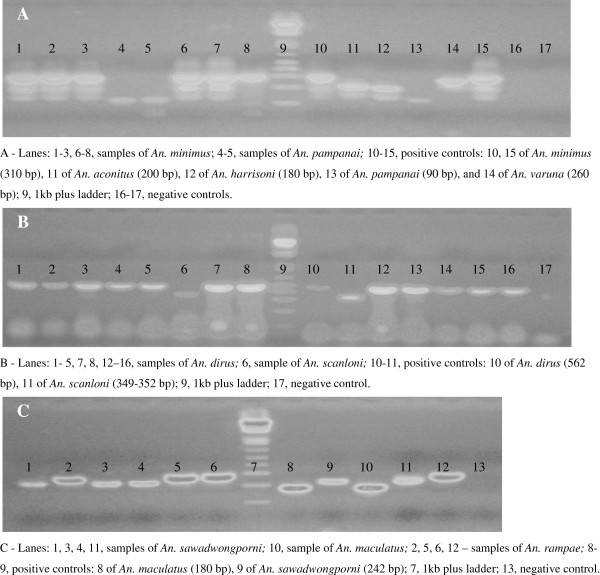
**Multiplex allele-specific PCR products on 2% agarose gel showing specimens of *An. pampanai *** **(A), *An. scanloni *** **(B) and *An. rampae *** **(C) collected in south-central Vietnam.**

All specimens belonging to the Minimus Complex (n = 83), Dirus Complex (n = 390) and Maculatus Group (n = 175) were processed for specific molecular identification. Misidentifications and discrepancies between morphological and molecular identifications occurred for specimens of each complex and group as shown in Table 2. Of the 83 specimens identified morphologically as *An. minimus*, five (6%) were *An. pampanai* based on molecular analysis (Figure 1A). Among the 390 specimens identified initially as *An. dirus*, DNA analysis showed that four (1%) were actually specimens of *An. scanloni* (Figure 1B). Finally, of the 175 specimens identified morphologically as *An. maculatus*, molecular identification revealed the presence of three species: 152 *An. maculatus* (88%), 16 *An. sawadwongporni* (9%) and six *An. rampae* (3%) (Figure 1C).

**Figure 2:**
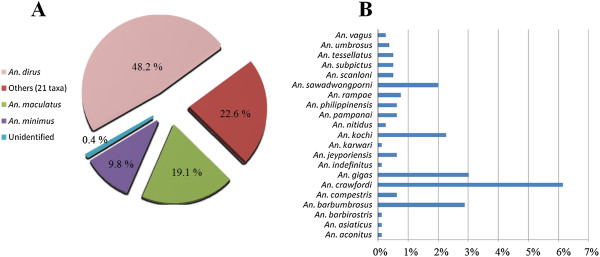
***Anopheles *species composition in south-central Vietnam based on molecular identification. (A)** Prevalence of the malaria vectors and other taxa; **(B)** percentage of the “other” 21 species displayed in **(A)** (arranged alphabetically from bottom to top).

In addition to the 20 species originally identified based on morphological characters, molecular identification revealed the presence of four other species, namely *An. scanloni, An. pampanai, An. sawadwongporni* and *An. rampae* (Table 2). The dominant species was *An. dirus* (48%), followed by *An. maculatus* (19%) and *An. minimus* (10%) (Figure 2). These three species are the main malaria vectors in Southeast Asia, and represented 77% of the *Anopheles* females captured in the study sites in Dak Nong and Binh Phuoc Provinces. The other 21 species (180 specimens) represented 23% of the total collection (Figure 2 A, B). Two specimens of the Dirus Complex and one of the Maculatus Group could not be identified by molecular methods (Figure 2). Of these 24 species, eight were commonly found in both provinces, whereas seven and nine species were only collected in Dak Nong or Binh Phuoc Province, respectively (Table 2).

Of the 311 specimens collected in Dak Nong Province, 308 were molecularly identified as members of 15 *Anopheles* species. *Anopheles maculatus* (48.7%) and *An. crawfordi* (15.9%) were the dominant species, followed by *An. dirus* (7.5%)*,* which is considered the main vector of “forest malaria”, *An. gigas* (8%), *An. barbumbrosus* (7%), *An. kochi* (5%) and nine other species (8%) – *An. asiaticus*, *An. campestris*, *An. nitidus*, *An. philippinensis*, *An. sawadwongporni*, *An. subpictus*, *An. tessellatus*, *An. umbrosus* and *An. vagus,* each of which was represented by 1–5 specimens (Table 3).

**Table 3:** **Specimens of the *Anopheles *** **species (n = 24) collected in Dak Nong and Binh Phuoc Provinces, Vietnam**

**Species**	**Provinces**	
	**Dak Nong**	**Binh Phuoc**
*An. aconitus*		1
*An. asiaticus*	1	
*An. barbirostris*		1
*An. barbumbrosus*	22	1
*An. campestris*	5	
*An. crawfordi*	49	
***An. dirus***	23	361
*An. gigas*	24	
*An. indefinitus*		1
*An. jeyporiensis*		5
*An. karwari*		1
*An. kochi*	16	2
***An. maculatus***	150	2
***An. minimus***		78
*An. nitidus*	1	1
*An. pampanai*		5
*An. philippinensis*	4	1
*An. rampae*		6
*An. sawadwongporni*	3	13
*An. scanloni*		4
*An. subpictus*	1	3
*An. tessellatus*	4	
*An. umbrosus*	3	
*An. vagus*	2	
Unidentified	3	
	311	486
Total	797	

Major malaria vector species are indicated in boldface.

Unlike Dak Nong Province, *An. dirus* (74%) and *An. minimus* (16%) accounted for 90% of the collections made in Binh Phuoc Province. These two species are known to be the main malaria vectors. *Anopheles sawadwongporni*, considered to be a secondary vector, represented 3% of the collections. Of the 17 species (486 specimens) collected in the province, 14 species (*An. aconitus*, *An. barbirostris*, *An. barbumbrosus*, *An. indefinitus*, *An. jeyporiensis*, *An. karwari*, *An. kochi*, *An. maculatus*, *An. nitidus*, *An. pampanai*, *An. philippinensis*, *An. rampae*, *An. scanloni* and *An. subpictus*) comprised only 7% of the collections, each represented by six or fewer specimens (Table 3). *Anopheles scanloni* and *An. rampae* were only collected in Binh Phuoc Province. This is the first discovery of *An. scanloni* in Vietnam and the first time *An. rampae* has been found so far south in the country. Comparative analysis of DNA sequences from these two species showed a high degree of similarity with sequences of these species recorded in GenBank (http://www.ncbi.nlm.nih.gov/). The sequences for the Vietnamese species are deposited in GenBank under accession numbers KJ746968-KJ746995.

### Detection of *Plasmodium* species and *Wuchereria bancrofti*

**Figure 3:**
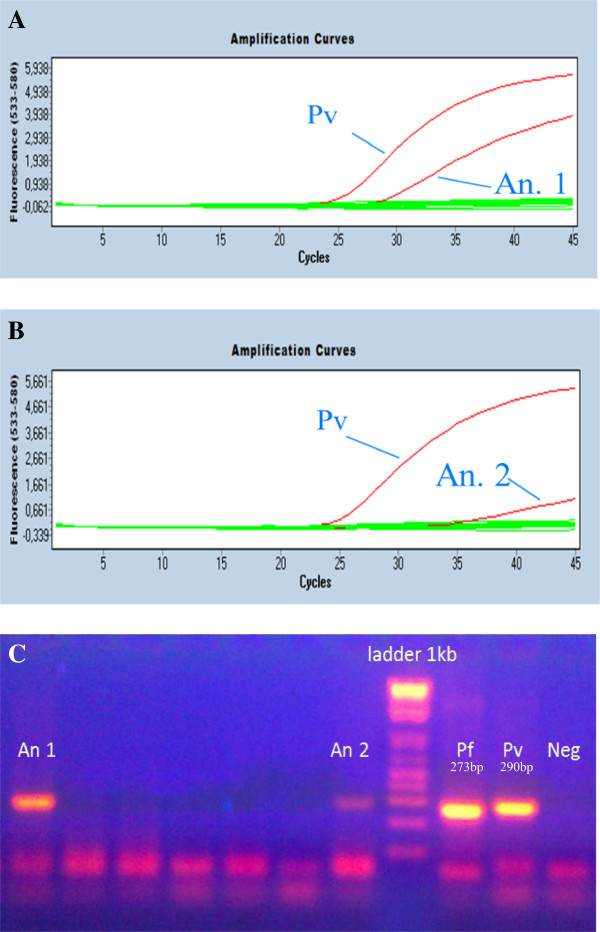
**Amplification curves in the multiplex real-time PCR for two mosquitoes positive for *P. vivax*. (A)** An. 1, *Anopheles dirus*; **(B)** An. 2, *Anopheles pampanai*; and **(C)** amplification of cytochrome oxidase cox I fragment by conventional PCR of *P. vivax* detected in *Anopheles* females collected in Vietnam. Pf and Pv = positive controls for *P. falciparum* and *P. vivax*.

A total of 765 *Anopheles* females were assayed for the presence of *Plasmodium* and *W. bancrofti* using a multiplex real-time PCR method with Taqman probes. One female of *An. dirus* and one of *An. pampanai* captured in Binh Phuoc Province were found to be infected with *P. vivax* (Figure 3A, B). The conventional PCR method of Cunha et al. [41] for parasite detection was used to confirm the infection of *P. vivax* (Figure 3C). The PCR products of the two *P. vivax* isolates were sequenced and both sequences detailed in Additional file 1. Based on these results, the *P. vivax* infection rate in *Anopheles* specimens collected in Binh Phuoc was 0.41% (2/486): 0.28% (1/361) for *An. dirus* and 20% (1/5) for *An. pampanai* (this high rate is due to the small number of specimens assayed and probably does not reflect the actual rate of infection in this species). None of the *Anopheles* specimens collected in the two provinces were infected with *P. falciparum*, *P. knowlesi* or *W. bancrofti*, and none collected in Dak Nong Province were infected with *P. vivax*.

## Discussion

### Diversity of *Anopheles* mosquitoes in Dak Nong and Binh Phuoc Provinces

Vietnam, and Southeast Asia in general, is known to have the most diverse *Anopheles* fauna and the largest number of species complexes and groups anywhere in the world [49-51]. *Anopheles dirus* and *An. minimus* are recognized as the main vectors of malaria in Southeast Asia, but other species have been incriminated, including *An. maculatus*, *An. sawadwongporni*, *An. sinensis*, *An. aconitus*, *An. pampanai*, *An. harrisoni*, *An. peditaeniatus* and *An. philippinensis*[1,3-5,7,52]. The density of each species depends on the ecology of the area where transmission occurs [53-56]. One of the sibling species of the Dirus Complex, *An. scanloni*, is considered to be one of the primary vectors in southern Thailand, and it has also been reported in southern Myanmar, but until now it has not been found in other countries [8,51,57]. In Thailand, *An. scanloni* is considered to have a non-continuous distribution because it is closely linked to limestone karst habitat [58], which has not been found in Vietnam (CTN, personal observation). Of the 390 females of the Dirus Complex included in the present study, four were molecularly identified as *An. scanloni*, the first record of this species in Vietnam. Of the eight species that comprise the Dirus Complex, three are now known to occur in Vietnam, i.e. *An. scanloni*, *An. dirus* and *An*. aff. *takasagoensis*[59]. Although *An. rampae* of the Maculatus Group has been recorded in northern and north-central provinces of Vietnam (Lang Son, Ninh Binh, Nghe An and Quang Binh Provinces) [40,60], this is the first discovery of this species in the south-central region of the country. This species, *An. maculatus* and *An. sawadwongporni* are three species of the Maculatus Group that are known to occur in Vietnam. The Maculatus Group includes nine species in southern Asia that are difficult to distinguish morphologically due to overlapping anatomical characters [49], but despite this shortcoming they are considered to play a role in malaria transmission in several regions [51,61-63]. *Anopheles maculatus* and *An. sawadwongporni*, for example, have been implicated in malaria transmission in Vietnam [1,5]. Although four and six specimens of *An. scanloni* and *An. rampae*, respectively, were collected in the present study, this once again demonstrates the importance of accurately identifying *Anopheles* species, especially sibling species, and evinces the reliability of molecular assays for this purpose. This result also highlights the need for further studies to elucidate the occurrence and role of these two species in malaria transmission in Vietnam.

In addition to *An. scanloni* and *An. rampae*, we collected 22 species of *Anopheles* that were previously known to occur in Vietnam [1,3-5,53,64-66]. The *Anopheles* species composition in Dak Nong and Binh Phuoc Provinces included common species, but also species specific to each province. This species specificity is most likely linked to the ecology of the study sites. These two adjoining provinces, with similar climatic conditions, favor the presence of eight common *Anopheles* species. However, Dak Ngo commune in Dak Nong Province is mainly covered by secondary forest and large plantations of coffee, pepper, cashew nuts or cassava, with people living nearby these plantations to manage their crops. Many insecticides (agrochemicals) were administrated to protect the farm products and dams were constructed for irrigation (CTN, personal observation). These activities are resulting in ecological changes that may affect the distribution of *Anopheles* species. In contrast, the study area of Bu Gia Map commune in Binh Phuoc Province is mostly covered by primary forest with natural and shaded streams, and this undisturbed tropical forest is known to favor the occurrence of primary malaria vectors, such as species of the Dirus Complex [2,4,55,67]. The distinctive environmental conditions of the two provinces influence the species composition of anophelines: *An. dirus* and *An. minimus* are the dominant species in Binh Phuoc Province whereas *An. maculatus* is dominant in Dak Nong Province. The presence of *An. dirus* s.l. and *An. maculatus* s.l is significantly correlated with sampling sites (X^2^ = 413.6, X^2^ lim = 3.84, P < 0.00001). However, the objective of this study was not to examine the link between ecological conditions and *Anopheles* species composition, therefore further study is needed to confirm these results.

### *Anopheles* vectors and transmission of parasites

The occurrence of malaria and lymphatic filariasis, two of the most common mosquito-borne parasitic diseases, the agents of which may be transmitted by the same vectors, was reported in Vietnam in the 1950s and 1970s [68-70], but no more recent studies have been conducted. The WHO considered lymphatic filariasis to be one of six infectious diseases that could be eradicated, and the regular surveillance of the disease and its vectors is mandatory to reach this goal [33]. Of the 24 *Anopheles* species identified in Dak Nong and Binh Phuoc Provinces during the present study, nine species (*An. aconitus*, *An. barbirostris*, *An. dirus*, *An. maculatus*, *An. minimus*, *An. philippinensis*, *An. subpictus*, *An. tessellatus* and *An. vagus*) were previously considered to be potential vectors for co-transmission of *Plasmodium* and *Wuchereria*[24]. Two mosquitoes, one *An. dirus* and one *An. pampanai*, were found infected by *P. vivax* but none were found infected with *W. bancrofti*, *P. falciparum* or *P. knowlesi*. The overall infection rate in Binh Phuoc Province was 0.41% (2/486), 0.28% (1/384) for *An. dirus* and 20% (1/5) for *An. pampanai*, and nil in Dak Nong Province. These results are in agreement with those reported by Nguyen in 2006 [31], who conducted a study of lymphatic filariasis in Khanh Hoa Province (located in south-central Vietnam east of Dak Nong and Bonh Phuoc Provinces) and reported that 3.64% of the population suffered from Bancroftian lymphatic filariasis; however, none of 144 *An. vagus* dissected were infected with *W. bancrofti*. Vietnam is one of the countries that have been considered to be in the pre-elimination phase of controlling lymphatic filariasis since 2011, and this study contributes to the surveillance of mosquitoes that potentially carry the parasite.

Cases of malaria reported in Vietnam are mostly due to *P. falciparum* (63%). *Plasmodium vivax* accounts for almost all other cases (37%) as *P. malariae* and *P. ovale* are seldom recorded [71] and a few cases attributable to *P. knowlesi* have been reported in south-central Vietnam [22]. A high proportion of asymptomatic cases, sometimes more than 80%, have been found in some areas [72]. Recently, sporozoite positive *Anopheles* females have been reported in various northern, central and southern areas of Vietnam [1,3-5,7,53], with *An. dirus* and *An. minimus* being the main malaria vectors. Besides these primary vectors, published reports also indicate the possible role of other *Anopheles* species, including *An. pampanai*, in malaria transmission [3,5,7,23]. The *P. vivax* infection rate in Binh Phuoc Province (0.41%; 2/486) is higher than the infection rate in the *Anopheles* population of Khe Ngang in Quang Binh Province (0.24%; 9/3,770) [1] and in seven species of *Anopheles* from Vietnamese and Cambodian populations (0.08%; 13/16,160) assayed by Durnez et al. [52]. However, it was lower than the infection rates previously reported by Marchand et al. [7] for *An. dirus* in Khanh Phu of Khanh Hoa Province infected with *P. vivax* (0.76%; 43/5,663), Sanh et al. [4] for *An. dirus* in Dong Thong of Ninh Thuan Province infected with *P. falciparum* (0.67%; 1/149) and Trung et al. [53] for *An. minimus* in Lang Nhot of Khanh Hoa Province infected with *P. vivax* (0.83%; 3/361). However, the infection rate of *An. pampanai* in the present study was quite high (20%; 1/5), due to the paucity of specimens collected. *Anopheles pampanai* was incriminated as a vector of *P. vivax* in Vietnam prior to this study [5,52]. Durnez et al. [52] reported that 1.54% (1/65) of the *An. pampanai* females collected in Ninh Thuan Province were infected with *P. vivax.* As for the transmission of *P. knowlesi* in Vietnam, Marchand et al. [7] reported that 0.55% (31/5,663) of the *An. dirus* females collected in Khanh Phu of Khanh Hoa Province were infected with *P. knowlesi*. No *Anopheles* specimens were found to be infected by either *P. knowlesi* or *P. falciparum* in the present study; however, malaria cases are still reported in Dak Nong Province, and in a similar survey conducted in that province in 2007, using the conventional PCR method of Cunha et al. [41], we found that 2.03% (5/246) of the *Anopheles* females collected were infected with *P. vivax*: *An. minimus* (3/76) and *An. maculatus* (2/22) (CTN & SM, unpublished data). Since this is the first study to examine *Anopheles* for co-infections of *Plasmodium* and *W. bancrofti* in south-central Vietnam, further study on a larger scale is needed to elucidate the role that different *Anopheles* species may play in the transmission of these parasites.

## Conclusions

The *Anopheles* fauna of Dak Nong and Binh Phuoc Provinces comprises at least the 24 species that were collected and identified during the present study, including species found in both provinces and species specific to each province. The primary malaria vector, *An. dirus*, was found in both provinces and was the dominant species in Binh Phuoc Province. *Anopheles maculatus* was the dominant species in Dak Nong Province but was scarce in Binh Phuoc Province. *Anopheles minimus* was only encountered in Binh Phuoc Province. This is the first report of the occurrence of *An. scanloni* of the Dirus Complex in Vietnam, and the most southerly collection of *An. rampae* of the Maculatus Group made in Vietnam thus far. No infections of *P. falciparum*, *P. knowlesi* or *W. bancrofti* were detected in 765 *Anopheles* females assayed. One *An. dirus* and one *An. pampanai* collected in Binh Phuoc Province were infected with *P. vivax*, giving an infection rate of 0.28% (1/384) for *An. dirus*, 20% (1/5) for *An. pampanai* and an overall infection rate of 0.41% (2/486). Further study is needed to gain a better understanding of the vector status of different *Anopheles* species and their individual roles in malaria transmission in south-central Vietnam.

## Competing interests

The authors declare that they have no competing interests.

## Authors’ contributions

CTN designed the study, was involved in organising the fieldwork, participated in the field collections, conducted the analyses, interpreted the results, wrote and revised the manuscript. GD was involved in interpreting the results of molecular analyses and revising the manuscript. VS and DP were involved in organising the field work and revising the manuscript. HQL organised the field work and participated in the field collections. REH assisted with the identification of *Anopheles crawfordi*, reviewed the results and revised the manuscript. SM participated in the design of the study, interpretation of the results and supervised all stages of the work, including revising the manuscript. All authors read and approved the final manuscript.

## Supplementary Material

Additional file 1 **Sequences of *Plasmodium vivax *** **isolated in *Anopheles *** **mosquitoes from Binh Phuoc, Vietnam.** Click here for file
