# Supplementary material for: Diversity of Anopheles mosquitoes in Binh Phuoc and Dak Nong Provinces of Vietnam and their relation to disease
Source: Parasit Vectors. 2014 Jul 9;7:316. doi: 10.1186/1756-3305-7-316 (PMC4227083; doi:10.1186/1756-3305-7-316)
Supplement: Additional file 1 — Sequences of Plasmodium vivax isolated in Anopheles mosquitoes from Binh Phuoc, Vietnam. [file 1756-3305-7-316-S1.docx]

**Additional file 1**: Sequences of *Plasmodium vivax* isolated in *Anopheles* mosquitoes from Binh Phuoc, Vietnam.

> 185bp [organism=*Plasmodium vivax*] *Plasmodium vivax* isolated in Vietnam, mitochondrial gene (coxI), partial sequence V204

GAGCATTTTTTACATCTACAACTATATTAATATCTATACCTACTGGAACAAAAATATTTAATTGGATATGTACATATATGGGTAGTAATTTTGGTATAACTCATAGTTCATCTTTATTATCATTACTATTTATATGTACATTTACTTTTGGTGGTACTACAGGAGTAATATTAGGTAATGCAGCT

> 174bp [organism=*Plasmodium vivax*] *Plasmodium vivax* isolated in Vietnam, mitochondrial gene (coxI), partial sequence V370

AGAGCATTTTTTACATCTACAACTATATTAATATCTATACCTACTGGAACAAAAATATTTAATTGGATATGTACATATATGGGTAGTAATTTTGGTATAACTCATAGTTCATCTTTATTATCATTACTATTTATATGTACATTTACTTTTGGTGGTACTACAGGAGTAATATTA
